# Surgical approach and recurrence risk in struma ovarii: A retrospective and systematic analysis

**DOI:** 10.17305/bb.2024.11287

**Published:** 2024-10-20

**Authors:** Miao Ao, You Wu, Zhiping Huo, He Zhang, Wei Mao, Bin Li

**Affiliations:** 1 Department of Gynecology, National Cancer Center/National Clinical Research Center for Cancer/Cancer Hospital, Chinese Academy of Medical Sciences and Peking Union Medical College, Beijing, China

**Keywords:** Benign struma ovarii, malignant struma ovarii, treatment, recurrence, prognosis

## Abstract

Struma ovarii (SO) represents a rare subset of ovarian germ cell tumors, with approximately 5% transforming into malignant SO (MSO). This study retrospectively analyzed clinical data from 23 SO patients treated at the Cancer Hospital of the Chinese Academy of Medical Sciences between January 2013 and December 2023, including 17 benign SO and 6 MSO cases. Additionally, a systematic review of 164 cases of MSO confined to the ovary, reported in the literature from 1946 to 2024, was conducted. Data on pathological type, treatment, and prognosis were extracted, and univariate and multivariate Cox regression analyses were performed to identify risk factors for recurrence in stage I MSO. The median age at diagnosis was higher for benign SO compared to MSO (58 vs 42.5 years), with 70.6% of patients being postmenopausal. Benign SO commonly presented with abdominal distension or mass, with more than half having ascites, while MSO patients were asymptomatic and lacked ascites. Cox regression analyses revealed that ovarian cystectomy was adversely associated with recurrence risk in stage I MSO, likely due to surgically induced capsular rent and potential tumor spillage. Significantly lower recurrence risks were observed in patients who underwent unilateral or bilateral salpingo-oophorectomy (HR ═ 0.36, *P* ═ 0.019; HR ═ 0.19, *P* ═ 0.004, respectively). This study highlights the importance of the surgical approach in the management of stage I MSO. A thorough preoperative discussion of the benefits and risks of different surgical approaches is recommended for patients desiring fertility preservation. Postoperative adjuvant therapy has not been shown to have a significant impact on prognosis. For the treatment of recurrent MSO, selecting appropriate surgical and adjuvant therapeutic strategies is essential to improve the long-term prognosis of MSO patients.

## Introduction

Struma ovarii (SO) is a rare and highly specific type of differentiated ovarian teratoma, classified as a special subtype of germ cell tumors, accounting for approximately 2%–3% of ovarian teratomas [1]. While most cases of SO are benign, around 5% may progress to malignant SO (MSO) [2]. The differentiation between benign SO and MSO is primarily based on pathological characteristics and biological behavior. Due to the rarity and often subtle clinical presentation of SO, distinguishing between benign and malignant forms prior to surgery can be challenging.

The primary treatment for SO is surgical resection. However, given the low incidence of MSO and the fact that most studies are limited to individual case reports or small case series, there are no established guidelines for the optimal extent of surgical resection [3]. Additionally, while the prognosis for MSO is generally favorable, the necessity for postoperative adjuvant treatment remains a topic of debate. Many experts recommend aggressive adjuvant therapies, such as total thyroidectomy, radioactive iodine (RAI) therapy, and thyroid hormone suppression therapy [4, 5]. On the other hand, some studies suggest that the malignant behavior of MSO is typically characterized by extra-ovarian spread or recurrent metastasis, and that over-treatment may be unnecessary for MSO confined to the ovary [6, 7].

In this study, we retrospectively analyzed the clinical data of 23 patients with SO admitted to our hospital over the past decade to compare the clinical manifestations and treatment modalities of benign SO and MSO. Additionally, we conducted a systematic review of 164 cases of MSO confined to the ovary reported in the literature over the past 80 years. By integrating this data with six cases of MSO treated at our hospital, we aim to summarize the pathological features, surgical approaches, adjuvant treatments, and prognoses for these patients, ultimately providing a reference for the clinical diagnosis and management of stage I MSO.

## Materials and methods

### Data collection and follow-up

This study involved 23 patients with SO who were treated at the Cancer Hospital of the Chinese Academy of Medical Sciences between January 2013 and December 2023. The inclusion criteria required patients to have undergone surgery with a pathologically confirmed diagnosis of SO, based on the World Health Organization (WHO) histological criteria. Diagnosis was confirmed by at least two pathologists, and patients were categorized into benign SO or MSO groups. The exclusion criteria were: (1) cases with incomplete clinical or follow-up data; (2) patients with significant comorbidities or other diseases that could affect survival outcomes; and (3) patients who were pregnant or lactating. Clinical data collected included age, menopausal status, body mass index (BMI), tumor location, tumor size, clinical symptoms, presence of ascites, and serum cancer antigen-125 (CA-125) levels, all obtained from the hospital’s electronic medical record system. Post-treatment, all patients were followed up by telephone, with the last follow-up recorded on December 31, 2023. The primary endpoints of the study were disease-free survival (DFS) and overall survival (OS). DFS was defined as the time period during which the patient remained free of disease following treatment, while OS was defined as the time from surgery to either death or the last follow-up for surviving patients.

### Literature review and data extraction

A literature search was conducted using the keywords “malignant struma ovarii” on PubMed (https://www.pubmed.gov) for publications between 1946 and 2024. Inclusion criteria for the literature review were: (1) histopathological confirmation of MSO; (2) tumor confined to the ovary, classified as stage I based on the FIGO 2014 staging system; and (3) availability of comprehensive clinical records, including surgical approaches and follow-up outcomes. Exclusion criteria included: (1) cases with extra-ovarian spread at initial diagnosis; (2) incomplete details on surgical intervention; and (3) reports lacking specific information on patient prognosis, including recurrence and survival outcomes. Data extracted from the literature included patient age, pathological characteristics, treatment methods, and survival outcomes, which were then analyzed further.

### Ethical statement

All patient-identifying information was kept confidential in accordance with the Declaration of Helsinki. The study received approval from the Institutional Review Board, and informed consent was waived due to the retrospective nature of the research.

**Table 1:** Clinical characteristics of 23 patients

**Characteristics**	**B-SO (*n* ═ 17)**	**MSO (*n* ═ 6)**	***P***
Age (years)	58 (16–65)	42.5 (28–62)	0.087
*Menopausal status*			
Yes	12 (70.6%)	1 (16.7%)	0.052
No	5 (29.4%)	5 (83.3%)	
BMI (kg/m^2^)	22.4 (17.4–27.9)	25.0 (17.7–30.9)	0.062
Tumor size (cm)	7.3 (3.7–25)	4.7 (3.1–15)	0.117
*Tumor site*			
Unilateral	17 (100.0%)	5 (83.3%)	0.261
Bilateral	0 (0%)	1 (16.7%)	
*Clinical symptoms*			
Bloating or finding abdominal masses	13 (76.5%)	2 (33.3%)	0.131
No symptoms	4 (23.5%)	4 (66.7%)	
*Ascites*			
With	9 (52.9%)	0 (0%)	0.048
Without	8 (47.1%)	6 (100.0%)	
*CA-125 levels*			
Elevated	8 (47.1%)	2 (33.3%)	0.660
Normal	9 (52.9%)	4 (66.7%)	

The Mann–Whitney *U* test was used for measurement data, and Fisher’s exact probability test was used to calculate the constitutive ratio for count data. Differences were considered statistically significant at *P* < 0. 05. B-SO: Benign struma ovarii; MSO: Malignant struma ovarii; BMI: Body mass index; CA-125: Cancer antigen-125.

### Statistical analysis

Statistical analysis was performed using Statistical Product and Service Solutions (SPSS) version 25.0 software. The Mann–Whitney *U* test was used to compare median values of continuous variables, while Fisher’s exact test was used for categorical variables. Survival analysis was conducted using the Kaplan–Meier method to generate survival curves, and the Log–Rank test was employed to assess differences in survival between groups. Univariate and multivariate Cox regression analyses were used to identify potential factors influencing recurrence in patients with stage I MSO. A *P* value of less than 0.05 was considered statistically significant for all tests.

## Results

### Clinical characteristics of benign SO and MSO

A total of 23 patients diagnosed with SO were included in the study, 17 of whom had benign tumors and 6 malignant tumors. Their clinical characteristics are detailed in Table 1. The median age of the benign SO patients was 58 years (range: 16–65 years), while the median age of MSO patients was 42.5 years (range: 28–62 years). Of the benign SO patients, 12 (70.6%) were postmenopausal, compared to only 1 (16.7%) MSO patient. The median BMI of benign SO patients was 22.4 kg/m^2^ (range: 17.4–27.9 kg/m^2^), whereas MSO patients had a median BMI of 25.0 kg/m^2^ (range: 17.7–30.9 kg/m^2^). The median tumor diameter was larger in benign SO cases, at 7.3 cm (range: 3.7–25 cm), compared to 4.7 cm (range: 3.1–15 cm) in MSO cases. MSO had an insidious onset and a higher proportion of asymptomatic cases compared to benign SO (62.5% vs 23.5%). Benign SO, on the other hand, was more likely to present with abdominal distension (5/17) or abdominal mass (8/17) as the first symptom. More than half of the benign SO patients (9/17) had ascites, whereas none of the MSO patients had ascites—a statistically significant difference (*P* ═ 0.048). Approximately 50% of benign SO patients had elevated serum CA-125 levels, compared to 33.3% (2/6) of MSO patients. In thyroid function tests, two benign SO patients showed mild preoperative elevation of free thyroxine (FT4), but no MSO patients exhibited thyroid abnormalities.

**Figure 1.**
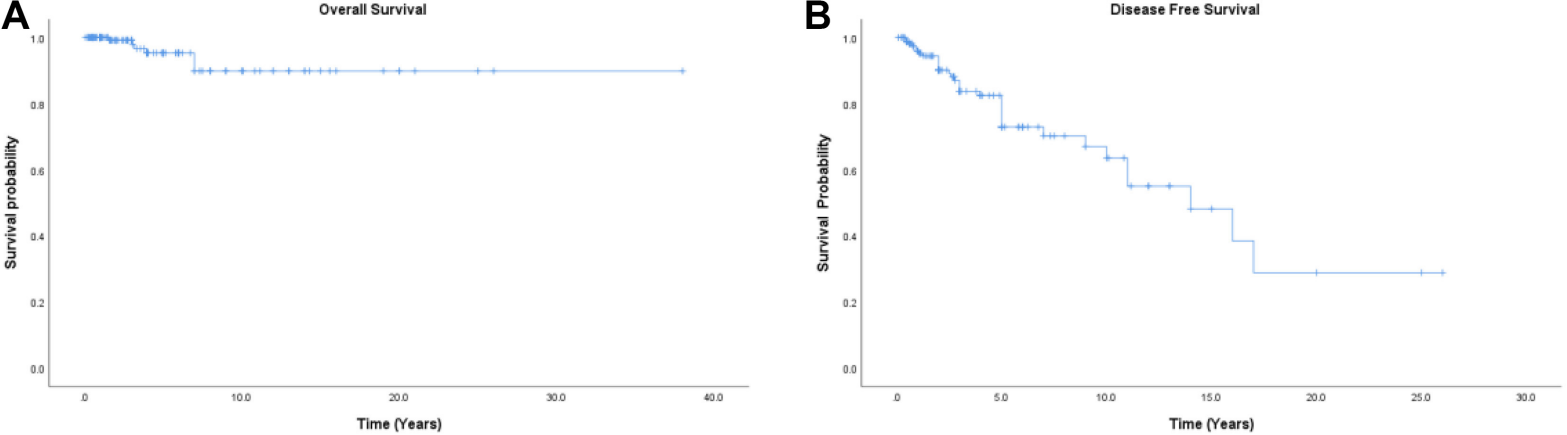
**Kaplan–Meier survival curves of MSO.** (A) The overall survival of all patients; (B) The disease-free survival of all patients. MSO: Malignant struma ovarii.

### Treatment and prognosis

Of the 17 benign SO cases, 5.9% (1/17) underwent ovarian cystectomy, 11.8% (2/17) underwent unilateral salpingo-oophorectomy, 11.8% (2/17) underwent bilateral salpingo-oophorectomy, and 70.5% (12/17) underwent total hysterectomy and bilateral salpingo-oophorectomy. The median survival time for benign SO patients was 66 months. Notably, the only recurrence occurred in the patient who had undergone ovarian cystectomy, who subsequently required a second surgical procedure (Table S1).

Of the six MSO cases, 16.7% (1/6) underwent ovarian cystectomy, 33.3% (2/6) underwent unilateral salpingo-oophorectomy with fertility preservation, and 50% (3/6) underwent total hysterectomy and bilateral salpingo-oophorectomy. Additionally, 66.7% (4/6) had concomitant major omentectomy, 16.7% (1/6) underwent appendectomy, and 33.3% (2/6) underwent lymph node dissection. Intraoperative cytology of peritoneal lavage fluid was negative for all six patients. Postoperative pathology revealed papillary carcinoma in five cases and mixed carcinoma (papillary carcinoma and poorly differentiated thyroid carcinoma) in one case, with all cases being stage IA. The median survival time for MSO patients was 87 months, with one recurrence 13 months after surgery in the patient with mixed carcinoma who had initially undergone ovarian cystectomy (Table S2).

**Table 2:** Pathologic features and treatment of 170 cases of stage I MSO

**Characteristics**	**Recurrence (*n* ═ 34)**	**Non-recurrent (*n* ═ 136)**	***P***
Age (years)	38.5 (33.8, 46.5)	46.0 (36.1, 56.0)	0.011
*Pathology*			
PTC	18 (53.0)	100 (73.5)	0.023
FTC	13 (38.2)	23 (16.9)	
Others	3 (8.8)	13 (9.6)	
*Adnexal surgery*			
OC	10 (29.4)	9 (6.6)	<0.001
USO	16 (47.1)	50 (36.8)	
BSO	8 (23.5)	77 (56.6)	
*With TH*			
N	25 (73.5)	72 (52.9)	0.030
Y	9 (26.5)	64 (47.1)	
*With TO*			
N	29 (85.3)	93 (68.4)	0.050
Y	5 (14.7)	43 (31.6)	
*With LN*			
N	31 (91.2)	112 (82.4)	0.208
Y	3 (8.8)	24 (17.6)	
*Adjuvant therapy*			
None	28 (82.3)	93 (68.4)	0.011
Chemotherapy	4 (11.8)	4 (2.9)	
Radiotherapy	0 (0)	1 (0.7)	
RAI	2 (5.9)	38 (28.0)	

OC: Ovarian cystectomy; USO: Unilateral salpingo-oophorectomy; BSO: Bilateral salpingo-oophorectomy; TO: Omentectomy; TH: Total hysterectomy; APP: Appendectomy; LN: Lymph node dissection; PTC: Papillary thyroid carcinoma; FTC: Follicular thyroid carcinoma; MSO: Malignant struma ovarii; RAI: Radioactive iodine.

### Analysis of factors associated with MSO recurrence

A total of 170 patients with MSO confined to the ovary were included in the study (164 reported in the literature and 6 from our center), with a median follow-up period of three years (ranging from 1 month to 38 years—Table S3). Tumor recurrence occurred in 20.0% (34/170) of patients. The mean OS for all patients was 37.0 years (95% CI: 31.95–37.40) (Figure 1A). The 5-year and 10-year DFS rates were 73.0% and 63.5%, respectively, with a mean DFS of 14.2 years (95% CI: 11.19–17.33) (Figure 1B). Pathologic characteristics and treatment approaches for recurrent and non-recurrent patients are detailed in Table 2. The median age of disease onset for recurrent patients was 38.5 years, which is younger than for non-recurrent patients. Postoperative pathology showed that 52.9% of recurrent patients had papillary carcinoma, 38.2% had follicular carcinoma, and 8.8% had mixed carcinoma. In terms of the initial surgical approach, 29.4% of recurrent patients had undergone ovarian cystectomy, 47.1% unilateral salpingo-oophorectomy, 23.5% bilateral salpingo-oophorectomy, and only 26.5% had undergone hysterectomy. Additionally, 14.7% of recurrent patients had undergone major omentectomy, and 8.8% had undergone lymphadenectomy. Most (82.3%) received no postoperative adjuvant therapy, while 11.8% received chemotherapy and 5.9% received RAI.

**Figure 2.**
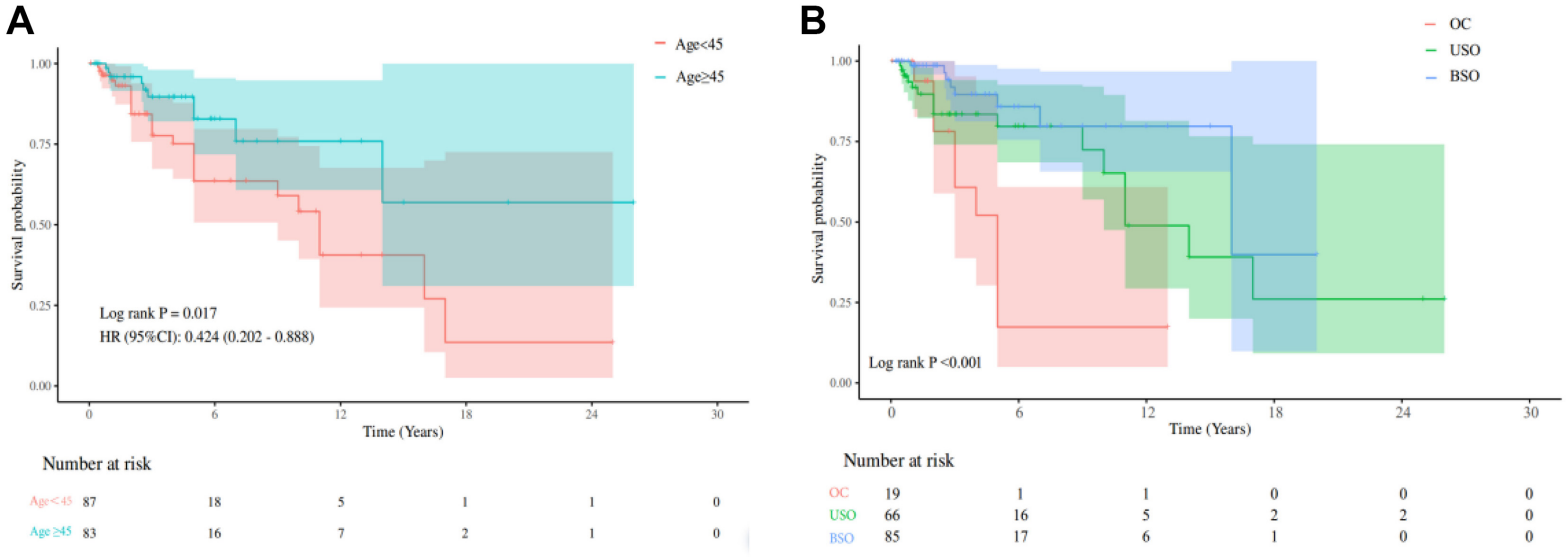
**Representative Kaplan–Meier survival curves of DFS.** (A) The age ≥ 45 group had better DFS than the age < 45 group; (B) The BSO group and the USO had better DFS than the OC group. DFS: Disease-free survival; BSO: Bilateral salpingo-oophorectomy; USO: Unilateral salpingo-oophorectomy; OC: Ovarian cystectomy.

Kaplan–Meier survival analysis showed that patients aged 45 years and older had significantly better DFS compared to younger patients (*P* ═ 0.017) (Figure 2A). Further analysis revealed that patients who underwent bilateral or unilateral salpingo-oophorectomy had better DFS than those who underwent ovarian cystectomy (*P* < 0.001) (Figure 2B). Univariate Cox regression analysis indicated that patients aged 45 and older had significantly improved DFS compared to younger patients (HR ═ 0.42, 95% CI: 0.20–0.89, *P* ═ 0.023). Moreover, adnexal surgeries, such as unilateral salpingo-oophorectomy (HR ═ 0.34, 95% CI: 0.15–0.77, *P* ═ 0.010) and bilateral salpingo-oophorectomy (HR ═ 0.16, 95% CI: 0.06–0.41, *P* < 0.010) were associated with significantly better DFS than ovarian cystectomy. Multivariate Cox regression analysis confirmed adnexal surgery as an independent factor influencing DFS, with a significantly lower risk of recurrence for patients undergoing unilateral salpingo-oophorectomy (HR ═ 0.36, 95% CI: 0.16–0.85, *P* ═ 0.019) or bilateral salpingo-oophorectomy (HR ═ 0.19, 95% CI: 0.06–0.59, *P* ═ 0.004) compared to those undergoing ovarian cystectomy (Table 3).

**Table 3:** Univariate and multivariate Cox regression analyses of DFS

**Factors**		**Univariate**		**Multivariate**	
		***P***	**HR (95% CI)**	***P***	**HR (95% CI)**
Age (years)	<45		Ref.		Ref.
	≥45	**0.023**	0.42 (0.20–0.89)	0.563	0.77 (0.32–1.87)
Pathology	PTC		Ref.		
	FTC	0.145	1.71 (0.83–3.50)		
	Others	0.865	1.11 (0.33–3.80)		
Adnexal surgery	OC		Ref.		Ref.
	USO	**0.010**	0.34 (0.15–0.77)	**0.019**	0.36 (0.16–0.85)
	BSO	**< 001**	0.16 (0.06–0.41)	**0.004**	0.19 (0.06–0.59)
With TH	N		Ref.		
	Y	0.280	0.66 (0.30–1.41)		
With TO	N		Ref.		
	Y	0.168	0.51 (0.20–1.33)		
With LN	N		Ref.		
	Y	0.279	0.52 (0.16–1.71)		
Adjuvant therapy	None		Ref.		
	Chemotherapy	0.393	1.58 (0.55–4.55)		
	Radiotherapy	0.985	0		
	RAI	0.149	0.34 (0.08–1.46)		

DFS: Disease-free survival; TO: Omentectomy; TH: Total hysterectomy; LN: Lymph node dissection; OC: Ovarian cystectomy; USO: Unilateral salpingo-oophorectomy; BSO: Bilateral salpingo-oophorectomy; PTC: Papillary thyroid carcinoma; FTC: Follicular thyroid carcinoma; RAI: Radioactive iodine.

Among the 34 patients with recurrent MSO, 11 had local recurrence, 11 had distant metastasis, and 12 had both local and distant recurrences. For recurrence treatment, 58.8% underwent secondary tumor cytoreduction, 26.5% received RAI, 5.9% received chemotherapy, 2.9% received targeted therapy, and 5.9% received no treatment.

## Discussion

SO is a rare ovarian germ cell tumor, first described in 1889. It is predominantly benign, with less than 5% transforming into MSO [8, 9]. In this study, we analyzed the clinical differences between benign SO and MSO in 23 patients. We found that the age of onset for both benign SO and MSO was primarily between 40 and 50 years. The median age of benign SO patients was older than that of MSO patients (58 years vs 42.5 years). Although a high proportion of postmenopausal women was noted, no statistically significant difference was observed between the two groups. Ryu et al. reported a median onset age of 48 years for MSO, similar to our findings, but in our study, benign SO patients were older than those with MSO. This discrepancy may be due to the small number of benign SO cases in our study [3, 10].

The clinical presentations of benign SO and MSO are often nonspecific, as most patients show no overt symptoms. Some may present with abdominal distention or a pelvic mass. In this study, we found that MSO tumors were typically smaller and more likely to be unilateral, whereas benign SO tumors tended to be larger and were more frequently associated with abdominal distension or mass symptoms. This aligns with the understanding that MSO progresses more insidiously, making early detection challenging. Notably, more than half of the benign SO patients had abdominal effusion, known as Pseudo-Meigs syndrome, a condition observed in approximately 20% of cases in other studies [11, 12]. Li et al. [13] noted that a tumor diameter over 10 cm and a patient age ≥49 years increase the risk of ascites in benign SO. Elevated CA-125 levels are also strongly associated with ascites. In cases where benign SO is combined with ascites and elevated CA-125, patients are often misdiagnosed with ovarian malignancy preoperatively [14]. Therefore, intraoperative cryopathologic examination is crucial for improving diagnostic accuracy and avoiding overtreatment.

Preoperative thyroid function tests can suggest SO, but not all cases show abnormal thyroid function. The incidence of hyperthyroidism in SO patients is approximately 5%–15% [15, 16]. In this study, only two benign SO patients had slightly elevated FT4 levels without significant hyperthyroidism. In cases where SO is suspected, thyroid function tests and thyroid ultrasound are essential for detecting potential thyroid abnormalities early.

Imaging features of SO are not always characteristic enough to distinguish between benign and malignant lesions before surgery. In this study, we found that the misdiagnosis rate with computed tomography (CT) was significantly higher than with pelvic magnetic resonance imaging (MRI) (30.4% vs 4.4%). On CT, SO often appears as a low-density cystic lesion with varying solid components, leading to misdiagnosis of malignancy, especially when large amounts of peritoneal fluid are present. In contrast, MRI offers better soft tissue contrast, and the limited diffusion of the colloidal and solid components of the thyroid may be suggestive of MSO [17].

Treatment for benign SO depends on the patient’s age and reproductive needs. For premenopausal women, ovarian cystectomy or unilateral salpingo-oophorectomy is preferred. However, benign SO has a recurrence risk, primarily as peritoneal implantation, with an incidence of about 1.3% [18]. Robboy et al. [6] suggested that benign SO showing malignant behaviors (e.g., extramural ovarian expansion, recurrence, or metastasis) should be classified as MSO. Factors influencing benign SO recurrence include the surgical approach, tumor capsule integrity, patient age, and tumor size. In cases of recurrent benign SO, fertility-sparing surgery may be considered, depending on metastasis extent and patient preference. For example, in a case of recurrent benign SO associated with peritoneal implantation likely due to tumor rupture during the initial surgery, we removed only the peritoneal metastases while preserving the uterus and adnexa, thus maintaining fertility potential. This approach may be suitable when metastasis is confined and resectable without involving the reproductive system. In unresectable cases, RAI therapy may be a viable alternative [19].

Treatment for MSO remains controversial. Surgery is the primary treatment, and fertility-sparing surgery, such as unilateral salpingo-oophorectomy, is recommended for younger patients. Ovarian cystectomy alone carries a high risk of recurrence and is not recommended. For patients without fertility concerns, hysterectomy and bilateral salpingo-oophorectomy are advised. Whether staging procedures, such as omentectomy and lymph node dissection similar to those used for ovarian epithelial cancer, should be performed remains inconclusive. Most MSO patients present at stage I, and only 5%–6% develop extra-ovarian metastases [4, 20]. Thus, for MSO patients with disease confined to the ovary, treatment decisions should consider long-term survival and avoid overly aggressive surgery or excessive postoperative therapy.

In this study, 170 patients with stage I MSO were included. Univariate Cox regression analysis identified patient age and adnexal surgery as risk factors for recurrence. Multivariate analysis confirmed that adnexal surgery was independently associated with DFS, excluding age as a confounding factor. This may be due to younger patients’ preference for fertility-sparing surgery. In early-stage MSO, ovarian cystectomy should be avoided due to the thin capsule of MSO, which may rupture during the procedure [6]. Our findings also suggest that resecting the greater omentum and pelvic lymph nodes does not significantly impact DFS in early-stage MSO. Extensive surgical staging is usually reserved for cases with confirmed or suspected spread, rather than applied routinely.

DeSimone et al. [20] recommended postoperative adjuvant thyroidectomy and RAI as the first line of treatment, shown to reduce MSO recurrence. However, their study included only 24 patients, limiting the generalizability of their findings. In our study, we reported seven cases of MSO from our center and expanded the sample size through a comprehensive literature review. Postoperative adjuvant treatments included RAI (23.5%), systemic chemotherapy (4.7%), and external pelvic irradiation (0.6%), but their effects on DFS in stage I MSO were not statistically significant. This aligns with Marti et al. [21], who emphasized weighing the potential benefit of RAI in reducing recurrence against the risk of ovarian failure, especially in premenopausal patients. RAI is typically recommended only for high-risk patients with extra-ovarian metastases, positive margins, or lymph node involvement [22].

The recurrence rate of stage I MSO in this study was 20%, which may be higher than the actual rate due to the inclusion of more case reports focusing on recurrences. Nevertheless, our rate aligns with previously reported MSO recurrence rates of 7.5%–35% [3, 21, 23]. Papillary thyroid carcinoma (PTC) is the most common type of MSO, accounting for 73.5% of patients without recurrence. Early-stage surgical resection of PTC confined to the ovary is an effective treatment strategy [24]. However, further analysis revealed that patients with follicular thyroid carcinoma (FTC) had a higher recurrence rate compared to non-recurrent patients (38.2% vs 16.9%). This is consistent with Jean et al. [25], who found that FTC patients are at higher risk for metastasis and recurrence. Therefore, FTC patients may require more aggressive postoperative adjuvant therapy.

The use of molecular sequencing could further categorize MSO and guide treatment. For instance, MSO patients with BRAF mutations tend to have worse clinical outcomes [26]. Additionally, 10%–15% of PTC cases may show TERT promoter mutations, which are associated with poor response to RAI and a higher likelihood of distant metastasis [27, 28].

MSO has various recurrence patterns, including local recurrence and distant metastasis. Local recurrences occur primarily in the ovary, peritoneum, or greater omentum, with intra-abdominal implant metastasis being the most common. Distant metastases are mainly hematogenous, affecting organs, such as the liver (9/34) and lungs (7/34), while lymph node metastasis is rare, occurring in only four cases. Treatment for recurrence is based on multimodal approaches. For local recurrence, secondary tumor reduction surgery, including bilateral adnexal resection, is recommended when feasible, followed by RAI therapy. If surgery is not possible, RAI-based systemic therapy is advised [29].

In this study, we examined the clinical presentation, treatment modalities, and recurrence risk factors for benign SO and early-stage MSO based on our center’s cases and a literature review. Our findings highlight that ovarian cystectomy significantly increases the risk of recurrence in stage I MSO and should be avoided when possible. Conversely, postoperative adjuvant therapy did not show a measurable benefit for long-term DFS. These findings provide important guidance for managing early-stage MSO. However, several limitations must be acknowledged. First, the small sample size from a single center limits the generalizability of the findings, although the literature review helps mitigate this. Second, the retrospective design and reliance on literature data, which often focuses on more severe cases, may bias recurrence rate estimates. Finally, the lack of genetic profiling limits the understanding of molecular factors contributing to recurrence.

Future multicenter studies with larger sample sizes are needed to validate our findings and comprehensively assess the impact of different surgical strategies on long-term survival and quality of life in MSO patients. Moreover, future research should focus on precision medicine approaches, including genetic testing-based targeted therapies, to optimize treatment for recurrent MSO patients.

## Conclusion

In conclusion, a comparative analysis of the clinical characteristics and treatments of SO patients revealed that MSO tends to have a more insidious onset and is more challenging to identify preoperatively. Additionally, adnexal surgery was found to be the primary factor influencing prognosis in patients with MSO confined to the ovary. Ovarian cystectomy should be avoided, as it is associated with worse outcomes, and the use of postoperative adjuvant therapy does not appear to impact the prognosis in stage I MSO. Further large-scale prospective studies are necessary to confirm these findings.

## Supplemental data

Supplementary data are available at the following link: https://www.bjbms.org/ojs/index.php/bjbms/article/view/11287/3553

## Data Availability

The datasets used and/or analyzed in the current study are available from the corresponding author upon reasonable request.
